# Performance of two *Ips* bark beetles and their associated pathogenic fungi on hosts reflects a species-specific association in the beetle-fungus complex

**DOI:** 10.3389/fpls.2022.1029526

**Published:** 2022-11-22

**Authors:** Xia Shi, Jiaxing Fang, Huicong Du, Sufang Zhang, Fu Liu, Zhen Zhang, Xiangbo Kong

**Affiliations:** Key Laboratory of Forest Protection of National Forestry and Grassland Administration, Ecology and Nature Conservation Institute, Chinese Academy of Forestry, Beijing, China

**Keywords:** *Ips* bark beetle, associated fungi, host suitability, beetle/fungus complex, signal interaction

## Abstract

When *Ips* bark beetles invade and colonize the host plants, their associated pathogenic fungal partners are carried into the phloem of the host trees. Host trees are lethally attacked by the beetle-fungus complex and the collective damage severely limits forestry production worldwide. It is of great importance to verify whether bark beetles and their associated fungi show concordant performance in terms of biology, physiology, and biochemistry on host trees. In this study, the two *Ips* bark beetles *Ips typographus* and *Ips subelongatu*s (Coleoptera: Curculionidae, Scolytinae), their respective associated pathogenic fungi *Endoconidiophora polonica* and *Endoconidiophora fujiensis*, and their respective host plants *Picea jezoensis* and *Larix olgensis* were selected as test material. Cross-inoculation experiments were conducted indoors and outdoors to investigate the differences in reproduction and development of two beetles and infectivity of two fungi on two plants, as well as the differences in physiological responses of two plants to two fungal infections. The results showed that *I. typographus* and *E. polonica* had excellent host performance on *P. jezoensis*; however, neither successfully colonized and infected *L. olgensis*. In contrast, *I. subelongatus* and *E. fujiensis* showed strong host suitability on *L. olgensis* and some degree of suitability on *P. jezoensis*, although the host suitability of *P. jezoensis* for *E. polonica* was significantly higher than that for *E. fujiensis.* In addition, we found that the absolute amount of ergosterol accumulated on the lesion was positively correlated with lesion area. The ergosterol amount and lesion area were both strongly correlated with the release of host monoterpenes, but had no obvious correlation with the concentration of fungi-induced phenols on the lesion area and the side-chain oxidation of lignin in the xylem of the infected sites. Based on these results, we confirmed that *“I. typographus*-*E. polonica*” and “*I. subelongatus-E. fujiensis*” complexes both showed the most suitable consistent performances on their own traditional hosts, establishing a stable species-specific association relationship in these two beetle-fungus complexes, with the “*I. subelongatus*-*E. fujiensis”* complex showing broader host suitability. From the perspective of physiological responses of plants to fungal infections, monoterpenes are an important indicator of host suitability.

## Highlights

• The host suitability of two *Ips* bark beetles and their associated pathogenic fungi was confirmed for the first time.
• The “*I. subelongatus*-*E. fujiensis”* complex shows a broader host suitability potential.
• The species-specific association between bark beetle and pathogenic fungus was verified.

## Introduction

Bark beetles (Coleoptera: Curculionidae, Scolytinae) are widespread in nature and more or less associated with relatively pathogenic fungi that promote successful colonization and development of the bark beetle on host plants (Six and Wingfield, 2011; Cale et al., 2017; Zhao et al., 2019). The use of artificial fungal inoculations provides evidence that pathogenic fungi are capable of killing mature trees (Solheim and Safranyik, 1997; Yamaoka et al., 1998, 2000; Kim et al., 2008), while in some cases bark beetles can successfully colonize trees in the absence of pathogenic fungi (Hetrick, 1949; Bridges et al., 1985; Jankowiak, 2005). It has been reported that the number of inoculations with pathogenic fungi required to kill a tree is approximately equal to the number of beetle attacks required to kill a tree (Christiansen, 1985a; Christiansen, 1985b; Franceschi et al., 2005). In general, the complex of bark beetle and pathogenic fungus would increase the overall strength of the attack and act synergistically to overcome the host’s strong defenses (Franceschi et al., 2005). Although some examples of bark beetles that have expanded their range and developed on novel hosts have been reported (Cullingham et al., 2011; Rosenberger et al., 2017), it remains unclear whether host suitability of bark beetles and pathogenic fungi is species-specific.

Trees have evolved defenses to inhibit or repel bark beetles and kill or stop the growth of their pathogenic fungal partners (Lewisohn et al., 1991; Woodward, 1992; Oven and Torelli, 1994). Among chemical defenses, oleoresins and phenols are the best studied (Ullah et al., 2021). Monoterpenes, the most conspicuous and well-studied constituents of conifer oleoresins (Franceschi et al., 2005), are an important means of chemical defense in trees (Raffa et al., 2005), and their release in phloem tissue can increase rapidly following bark beetle or pathogenic fungal attack. Phenols are also associated with resistance of conifers to bark beetles and their associated fungi (Lieutier et al., 1991; Ralph et al., 2006). Pathogenicity may allow the fungus to survive in a living tree until the tree’s defenses are weakened and the environment becomes more favorable for growth (Six and Wingfield, 2011). Lesion length is used to assess fungal colonization of the phloem of living trees, and longer lesions are thought to be more pathogenic to hosts (Krokene and Solheim, 1996; Yamaoka, 2017) and thus of greater benefit to bark beetles by weakening tree defenses and preventing the host from developing its potential resistance (Solheim and Safranyik, 1997; Franceschi et al., 2005; Zhao et al., 2019).

Although beetles and pathogenic fungi share common advantages in overcoming host defenses, they also require sufficient nutrients to develop rapidly in trees. However, some of the nutrients required by beetles are not present in hosts and are only available in fungi, such as ergosterol (Guevara-Rozo et al., 2020). It is required by beetles for ovogenesis and normal larval development and pupation (Bentz and Six, 2006; Janson et al., 2008). Ergosterol has also been used as a proxy to determine fungal biomass in previous studies (Nylund and Wallander, 1992; Guevara-Rozo et al., 2020). Although the host tree is rich in nutrients, it is not very accessible to beetles and fungi. The nutrient-rich inner bark and cambium can be physically protected by lignin, and the nutrient quality of the bark can also be reduced by lignin, which provides the physical defense to protect nutrients from exploitation by bark beetles (Wainhouse et al., 1990). There are reports that white-rot Basidiomycota associated with bark beetles can degrade lignin (Bugg et al., 2011; Six and Elser, 2019; Six, 2020); whether pathogenic fungal partners degrade lignin structure to destroy host physical defenses remains unclear. Most bark beetles cause damage to Pinaceae species that contain only guaiacyl (G)-lignin, with the β-O-4-aryl ether bond being the dominant structural linkage in lignin dimers (Adler, 1977). The selectivity of the thermochemolysis process with tetramethylammonium hydroxide (TMAH) for the β-O-4 bond allows the degradative change of the lignin side chain to be monitored (Hatcher and Minard, 1996; Mckinney and Hatcher, 1996; Filley et al., 2000). The ability to infer the relative degradation state of the propyl side chain is achieved by using molecular ratios such as the 3,4-dimethoxybenzoic acid (G6) to 3,4-dimethoxybenzldehyde (G4) parameters, and the increase in G6/G4 indicates a higher degree of side chain oxidation reaction in the lignin degradation sample (Hatcher and Minard, 1996; Filley et al., 2000; Geib et al., 2008).

*Ips typographus* L. and *I. subelongatus* Motschulsky are the main pests responsible for significant mortality in coniferous forests in the northeastern region of China (Gao et al., 2000; Zhou et al., 2011; Chen et al., 2015; Fang et al., 2020a, 2020b), with *I. typographus* causing severe damage to Yezo spruce (*Picea jezoensis*) and *I. subelongatus* killing many Changbai larch (*Larix olgensis*). The two bark beetles have similar morphological characteristics (Shi et al., 2020, Shi et al., 2021) and are genetically very closely related (Wang et al., 2021; Du et al., 2021; Du et al., 2022), but their major host plants are different, e.g., spruce (*Pices* spp.) for *I. typographus* and larch (*Larix* spp.) for *I. subelongatus* and sometimes pine (*Pinus* spp.) (Xiao, 1992). Like most bark beetles, these two *Ips* species are associated with many fungal species, especially pathogenic fungi. The highly pathogenic fungi associated with two bark beetles both belong to the genus *Endoconidiophora* (formerly *Ceratocystis*), whose species are always the first to invade sapwood, spread sufficiently, and sporulate rapidly (Paine et al., 1997). *Endoconidiophora polonica* and *E. fujiensis* are associated with *I. typographus* and *I. subelongatus*, respectively, and were not found on each other’s vectors (summarized in **Table 1**, Viiri and Lieutier, 2004; Jankowiak, 2005; Jankowiak et al., 2009; Persson et al., 2009; Yamaoka et al., 2009; Kirisits, 2010; Meng, 2013; Repe et al., 2013; Chang et al., 2019; Wang et al., 2020), thus appear to be relatively vector-specific. The “*E. polonica*-*I. typographus*” and “*E. fujiensis*-*I. subelongatus*” complexes provide suitable models to study the performance of bark beetles and associated fungi to tree hosts.

**Table 1:** Selected information from research on *Endoconidiophora* fungi isolated from two *Ips* bark beetles.

Source of fungi	Statistical indicator of fungal isolates	*Endoconidiophora* spp.		Sampling area	References
		*E. polonica*	*E. fujiensis*		
*I. typographus*	total percentage of fungal isolates in number	7.0%	0	Northeastern China	Chang et al., 2019
*I. typographus*	frequency of occurrence of the fungus	22.0-50.0%	0	France	Viiri and Lieutier, 2004
*I. typographus*	frequency of occurrence of the fungus	3.7-12.7%	0	Southern poland	Jankowiak, 2005, Jankowiak et al., 2009
*I. typographus*	frequency of occurrence of the fungus	76.0-86.0%	0	Northeastern Poland	Kirisits, 2010
*I. typographus*	frequency of occurrence of the fungus	0.9%	0	Southern Sweden	Persson et al., 2009
*I. typographus*	frequency of occurrence on the beetles	2.6%	0	Slovenia	Repe et al., 2013
*I. subelongatus*	total percentage of fungal isolates in number	0	3.6%	Northeastern China	Wang et al., 2020
*I. subelongatus*	total percentage of fungal isolates in number	0	12.70%	Northeastern China	Meng, 2013
*I. subelongatus*	frequency of occurrence of the fungus	0	9.8-76.9%	Japan	Yamaoka et al., 2009

The objective of this study is to investigate the suitability of bark beetles and pathogenic fungi on host plants. This was done in two steps. In the first step, the physiological response of bark beetles and their associated pathogenic fungi on trees was tested to confirm their performance on host plants (direct pathway), including beetle development and fungal infestation on two host trees through artificial inoculation experiments. In a second step, the induced biochemical response of the pathogenic fungi to the host trees was evaluated to determine the specific “benefit” of the pathogenic fungi to the beetle vector (indirect pathway). Ergosterol production, monoterpene release, phenol induction and lignin side chain oxidation were tested as indicators of “benefit”. Our results may provide new insights into the infestation of host trees by the complex “*E. polonica*-*I. typographus”* and “*E. fujiensis*-*I. subelongatus*” and contribute to a better understanding of the complex interactions between insects and fungus-associated host plants.

## Materials and methods

### Experiments with artificial insect inoculation

#### Sampling of bark beetles

Overwintered *I*. *typographus* were collected in traps baited with synthetic aggregation pheromones at Jingouling Forest Farm (43°21’30”N 130°11’2”E; elevation=660m), Wangqing County, Jilin Province, China. Similarly, overwintered *I. subelongatus* were collected from Mengjiagang Forest Farm (46°20’16”N 130°32’42”E; elevation=360m), Huanan County, Heilongjiang Province. Beetles were collected daily and separated by sex as previously described (Schlyter and Cederholm, 1981; Chen, 2013). All beetles were used to inoculate tree trunks within two days of collection.

#### Inoculation of adult beetles into tree trunks

We compared the reproductive and developmental parameters of two *Ips* species with felled tree trunks. Trees of *P. jezoensis* and *L. olgensis* had a diameter at breast height of approximately 16 cm and were free of visible signs of insects or disease. In 2019 and 2021, trunks were taken from two host trees in a stand at Jingouling Forest Farm. Trials were initiated once in June during the short flight season of two *Ips* species. One tree of each species was felled in early June 2019 and another in early June 2021. Four stems (~50 cm long) were cut from each tree. Both ends of the stems were sealed with hot paraffin wax to preserve moisture. Twenty females and 20 males were placed on each stem within three days of felling the trees. Each stem was sealed separately in a stainless steel mesh bag (60 mesh, 0.1 mm wire diameter) at room temperature of ~25°C. To compare the invasion ability of two *Ips* species on two host trees, we cleaned only the dried and dead bark outside the trunk and didn’t punch any holes. Each *Ips* species was introduced into two stems per tree. After three weeks, we debarked each stem and counted the brood at each life stage. Developmental indices were recorded for successfully colonized adult females and their broods. Egg-laying gallery length, larval gallery length, and larval head capsule width were measured, and brood adults were weighed. The number of all eggs and incubated eggs was counted to calculate female fecundity and egg hatchability, respectively. For each treatment, one successfully reproductive adult female was taken as one biological replicate, and a total of 40 replicates were taken; one brood at the same developmental stage was randomly selected as one biological replicate, and a total of 60 replicates were taken.

### Experiments with artificial fungal inoculation

In early June 2021, we selected 12 healthy *P*. *jezoensis* trees and 12 healthy *L*. *olgensis* trees (i.e., no visible signs of biotic or abiotic stress above ground) from a forest stand at Jingouling Forest Farm. Trees were randomly assigned to one of three inoculation treatments: *E*. *fujiensis*, *E*. *polonica*, or 2% malt extract agar media (MEA) without any fungal culture as a control (CK-MEA), using three trees per treatment and the remaining three trees as non-inoculated controls (CK-healthy trees). Source data for *E*. *fujiensis* and *E*. *polonica* are listed in **Table 2**. A total of 15 inoculation sites were punched on each tree. All sites were spaced 1.0 m to 1.4 m above the ground and included three rows (i.e., 20 cm spacing between rows, 4 cm spacing within the same row). Two fungi grew at MEA at 23°C in complete darkness for 1 week before inoculation trials. In the field, cork borers (inner diameter 6 mm) were used to obtain culture plugs from the edge of the growing colony and cork plugs from the tree. The fungal plugs containing the agar inoculum were inserted into the tree hole with the mycelium facing the surface of the sapwood. All inoculation sites were sealed with sterile caps of plastic centrifuge tubes to prevent contamination with other microorganisms, and then wrapped around the trunk with plastic film to prevent moisture loss.

**Table 2:** Detailed source information on two *Endoconidiophora* fungi used in tree inoculations.

Fungal species	Beetle species/Location of isolation	Site name	Host	Collection year	Isolate no.	Latitude/longitude
*Endoconidiophora polonica*	*Ips typographus*/exoskeleton and gallery	Jingouling Forest Farm, Yanji City, Jilin Province	*Picea jezoensis*	2017	CFCC 53555	N:43°21’30”E:130°11’24”
*Endoconidiophora fujiensis*	*Ips subelongatus*/exoskeleton and gallery	Zhanhe Forest Farm, Yichun City, Heilongjiang Province	*Larix gmelinii*	2017	CXY1912	N:48°38’30”E:126°38’17”

CFCC, China Forestry Culture Collection Center; Beijing; China; CXY, the culture collection of the Chinese Academy of Forestry.

Three months after inoculation, phloem and sapwood tissue samples were collected from the lesions. After carefully removing bark from selected inoculation sites, we found that fungal inoculations produced much longer lesions than CK-MEA, indicating the success of our inoculations (Bonello et al., 2006; Cale et al., 2019; Guevara-Rozo et al., 2020). In each inoculated series, two complete lesions were completely removed from the trunk. On the CK-healthy trees, we collected two samples (4 cm × 5 cm) using similar sampling procedures as above. On each tree, we photographed the lesion on the phloem and later analyzed it using Image Pro Plus software (version 6.0.0.260, Media Cybernetics, Inc) to calculate the infested area. All tissue samples were stored in a biological sampling box (~4°C) and then transported to the laboratory in a freezer at –20°C.

#### Sampling and analysis of host tree volatiles

Host volatiles released from culture-inoculated trees and CK-healthy trees were collected one day post inoculation (1 dpi) and 91 days post inoculation (91 dpi). The dynamic headspace sampling system was used to collect the volatile samples. For each tree, the volatiles of the trunk were sampled at the inoculation sites. The sampling area for aeration was enclosed with a sheet of food-grade polyethylene (PE) (35 cm × 48 cm). One end of the sampling tube (6 mm inner diameter × 160 mm length) containing the Porapak-Q absorbent (i.e., 200 mg, 50–80 mesh in a Chrompack, CNW Technologies GmbH, Germany) was connected to the air inlet of the atmospheric sampler (QC-1S, Beijing Labor Protection Institute, China), and the other end was placed under the airtight PE film so that volatiles could be trapped in the Porapak-Q absorbent. Sampling took 20 min per tree at an airflow rate of 500 ml min^–1^. After sampling was completed, both ends of the sampling tube were quickly sealed with aluminum foil and parafilm and stored in a biological sampling box (4°C). Upon return to the laboratory, we eluted the absorbed chemicals with 12 ml hexane (HPLC grade) for monoterpene analysis. Porapak-Q sampling tubes were conditioned prior to volatile collection according to the methods used by Fang et al. (2020a).

Samples were analyzed using HP 6890 gas chromatograph (GC, Agilent Technologies, Palo Alto, CA, USA) equipped with a flame ionization detector (FID). A DB-5MS fused silica capillary column (30 m length × 0.25 mm inner diameter × 0.25 mm film thickness, J&W Scientific, Folsom, CA, USA) was used to identify and quantify host monoterpenes. Twenty microliters of 500 μl each of the conditioned aeration extract were concentrated to 1-2 μl under a gentle stream of nitrogen and then injected into the GC port (220°C) in splitless mode. Nitrogen was used as the carrier gas at a column head pressure of 13 psi. The GC oven was first held at 60°C for 1 min, then increased from 60 to 240°C at 6°C min^–1^, and finally increased from 240 to 280°C at 20°C min^–1^, where it was held for 5 min. The identity of the monoterpene peaks was confirmed by GC injection of authentic standards, while the absolute amounts were determined by comparing the peak areas with those of an external standard of heptyl acetate (10 ng/injection).

For further confirmation, a sample from each treatment was analyzed by GC-MS on a HP 7890 GC connected to a 5975 mass spectrometer (Agilent Technologies, Palo Alto, CA, USA; EI mode, 70 eV; mass range, 30-560 amu) on DB-5MS capillary columns whose programmed settings were the same as above. The injector, ion source, and transfer line temperatures were maintained at 220, 230, and 250°C, respectively. Mass spectra were recorded at a rate of five spectra per second after the 3 min solvent delay. Helium at 1.0 mL min^–1^ was used as the carrier gas. The GC capillary column and procedure settings were the same as for the GC analyses above. The compounds were identified by comparing the retention times and mass spectra with those of the authentic standards.

#### Extraction and analysis of ergosterol

We freeze-dried all tissue samples from each tree for three days, weighed the total phloem lesions, and finally ground them in liquid nitrogen. Both the dried phloem lesion samples and the phloem samples from healthy trees weighed 300 mg for ergosterol extraction, which was performed according to previously published methods (Guevara-Rozo et al., 2020) with slight modifications. Briefly, 5 ml of KOH (10% w/v) in methanol (HPLC grade) was added to the sample in a test tube. The tube was tightly sealed, sonicated for 15 min, and then heated to 70°C for 90 min. After cooling to room temperature, 1 ml of distilled water was added. An aliquot of 2.5 ml pentane was added to the sample, shaken for 1 min, then centrifuged at 1000 g min^–1^ and finally the top layer was transferred to a new test tube. The procedure was repeated one more time. All extracts were combined, evaporated to dryness, redissolved in 200 μl methanol, and finally filtered through glass wool. One microliter of the extract was injected into the GC-MS port (300˚C) equipped with a DB-5MS fused silica capillary column (see above), using pulsed splitless mode. Helium was used as the carrier gas at a flow rate of 1.5 ml min^–1^, with a temperature program of 50°C for 2 min, then 25°C min^−1^ to 240°C, and then 15°C min^–1^ to 300°C (held for 7 min). Due to the trace amount of ergosterol in the sample, full scan and selective ion monitoring modes were used simultaneously. Selective scanning ions *m/z* 337, 363, and 396 are used to confirm the presence of ergosterol based on abundance ratio and retention times. In addition, the absolute amount of ergosterol in the phloem lesion (µg) was determined using a calibration curve with dilutions of the standard in methanol at five dilutions ranging from 5, 10, 50, 100 to 500 µg ml^–1^. There were three biological replicates for each treatment.

#### Extraction and analysis of phenols

The extraction of phenols was performed according to the procedure described by Raffa et al. (2017) with some modifications. Briefly, 100 mg ± 2 mg of freeze-dried samples (culture-inoculated and healthy phloem) that had been grounded in liquid nitrogen were used to extract phenolic compounds. The sample was immersed in the tissue into 1 ml of methanol (HPLC grade), shaken for 15 sec and sonicated for 20 min. These mixtures were then left in the dark for 24 h, followed by centrifugation at 14000 rpm for 10 min. The supernatant was collected and transferred to 2 ml glass chromatography vials. The remaining solid was extracted a second time using the procedure described above, and the two extracts were combined and stored at –20°C until analysis.

The phenol extracts were detected and identified using Waters 2695 high-performance liquid chromatography (HPLC) (Waters Corporation, Milford, MA, USA) with a dual λ absorbance detector (Waters 2487). The injection volume was 10 μl. Separations were performed on a SunFire C18 column with 4.6 × 250 mm and 5 μm particle diameter (Waters, Milford, MA, USA). The autosampler temperature was 24°C, and the column temperature was 50°C. The binary mobile phase, 0.1% acetic acid in water (solvent A) and 100% methanol (solvent B), had a flow rate of 0.8 ml/min. The total run time was 45.0 min. The linear gradient was: 0.0, 69.0; 25.0, 35.0; 26.0, 0.0; 35.0, 0.0; 36.0, 69.0; 45.0, 69.0 [cumulative run time (min), % solvent A]. The compounds were detected at wavelengths of 280 nm and determined by comparing the retention times with chemical standards. Standard curves used to quantify phenolic compounds were calculated from dilutions prepared from analytical standards of (+)-catechin, quercetin, isorhamnetin, caffeic acid, taxifolin, *ρ*-coumaric acid, ferulic acid, resveratrol, piceid and pinoresinol. Final phenolic concentrations were expressed as milligrams of compound equivalent per gram of freeze-dried phloem (mg/g dw phloem).

#### TMAH thermochemolysis analysis

The lesion on the surface of the freeze-dried sapwood was carefully scraped with a blade and 5 mg weighed for TMAH thermochemolysis analysis, which was performed with some modifications according to Geib et al. (2008). Each sample and 150 μl TMAH (25 wt%) in methanol (Fisher Scientific) were placed together in a borosilicate grass tube (5 mm inner diameter × 150 mm length) and mixed for 30 sec. The methanol was evaporated at 75°C for 24 h. The tube was sealed with an alcohol burner using nitrogen as the inert gas and then baked at 240°C for 30 min. After the tube cooled to room temperature, it was frozen in liquid nitrogen to prevent product loss and broken open. The products were extracted from the tubes using ethyl acetate (HPLC grade). By washing the tube walls, all products (approximately 1 ml per sample) were filtered through glass wool, pooled, and concentrated to 100 μl under a gentle steam of nitrogen. The products were analyzed using the same equipment and methods described above for the analysis of host tree volatiles. Compounds were identified by comparing their retention times and mass spectra with commercial G4 and G6 standards in GC and GC-MS. Their quantitative analyses were performed by peak area comparisons with the external standard heptyl acetate (10 ng/injection). There were three biological replicates for each treatment.

### Chemicals

The chemicals *S*-(-)-α-pinene (ACROS, 98%, CAS: 7785-26-4), myrcene (ACROS, 90%, CAS: 123-25-3), *S*-β-pinene (ACROS, 98%, CAS: 18172-67-3), *S*-(-)-limonene (J&K, 96%, CAS: 5989-54-8), terpinolene (J&K, 95%, CAS: 586-62-9), 3-carene (ACROS, 90%, CAS: 13466-78-9), (±)-camphene (ACROS, 95%, CAS: 79-92-5), ergosterol (Sigma-Aldrich, CAS: 57-87-4), 3,4-dimethoxybenzldehyde (J&K, 99%, CAS: 120-14-9), 3,4-dimethoxybenzoic acid (J&K, 98%, CAS: 2150-38-1), caffeic acid (Sigma-Aldrich, 98%, CAS: 331-39-5) and *ρ*-coumaric acid (Sigma-Aldrich, 98%, CAS: 501-98-4) were purchased from J&K Co. Ltd (Beijing, China). (+)-Catechin (Aladdin, 97%, CAS: 154-23-4), quercetin (Aladdin, 95%, CAS: 117-39-5), isorhamnetin (Aladdin, 98%, CAS: 480-19-3), taxifolin (Aladdin, 98%, CAS: 480-18-2), ferulic acid (Aladdin, 99%, CAS: 1135-24-6) and resveratrol (Aladdin, 99%, CAS: 501-36-0) were purchased from Aladdin Co. Ltd (Shanghai, China). Piceid (MΛCKLIN, 97%, CAS: 65917-17-2) was purchased from MΛCKLIN Co. Ltd (Shanghai, China). Pinoresinol (bidepharm, 95%, CAS: 487-36-5) were purchased from bidepharmatech Co. Ltd (Shanghai, China).

### Statistical analyses

Data were tested for the assumptions of homoscedasticity and normality by using Kolmogorov-Smirnov tests. When necessary, data were transformed to Log(X+1) before analysis. Significant differences in developmental indices of each *Ips* species between spruce and larch were compared using a T-test for independent samples at the 0.05 level. At 91 dpi, significant differences in lesion areas, ergosterol, and G6 to G4 ratios induced by the same culture between two tree species were compared with a T-test for independent samples at the 0.05 level, and then significant differences between different cultures on the same tree were analyzed with a one-way ANOVA followed by a Turkey HSD test at the 0.05 level. Linear regression analysis was performed to investigate and visualize the relationships between lesion areas and absolute ergosterol levels. Significant differences in the release rates of monoterpenes induced between the MEA culture, the fungal culture at 1 dpi, and the fungal culture at 91 dpi on the same tree were also compared using a T-test for independent samples at the 0.05 level. Significant differences in phenols induced on each tree between culture inoculation treatments were analyzed using a one-way ANOVA followed by a Turkey HSD test at the 0.05 level. The correlation between tree biochemical responses (monoterpene release, phenol induction and ratio of G6 to G4) and fungi (lesion areas and absolute ergosterol amount) at 91 dpi was analyzed using Pearson correlation at the 0.05 level. IBM SPSS Statistics for Windows 23.0 (IBM Corp.) and GraphPad PRISM 9.0.0 (Graphpad Software Inc.) were used for statistical procedures and chart generation.

## Results

### Developmental indices of bark beetles in response to wood inoculation

**Figure 1:**
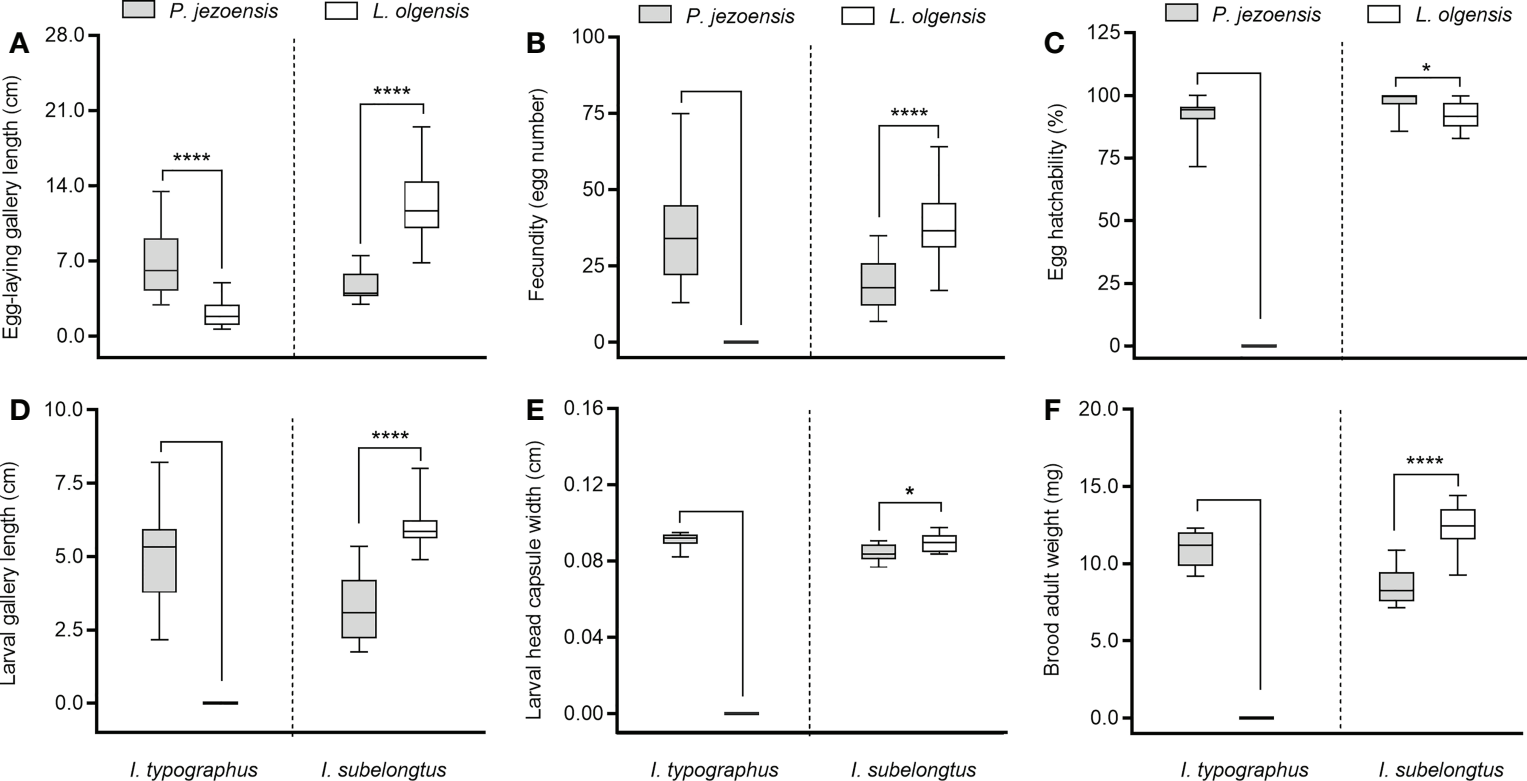
Minimum to maximum developmental indices of *I typographus* and *I subelongatus* in response to stem inoculation. **(A)** Egg-laying gallery length of female; **(B)** female fecundity (number of eggs); **(C)** egg hatchability (%); **(D)** larval gallery length; **(E)** larval head capsule width; **(F)** brood adult weight. Asterisks indicate significant differences (*p< 0.05, ****p < 0.0001; T-test for independent samples, *α*=0.05).

Overall, the two *Ips* species developed differently on *L*. *olgensis* and *P*. *jezoensis* stems. *Ips typographus* was well developed on *P. jezoensis* and showed a complete developmental generation; however, on *L*. *olgensis*, females tunneled only short galleries (< 2.5 cm) and did not lay eggs (**Figures 1A, B**), so no data on brood development index were available (**Figures 1D–F**). In contrast, *I*. *subelongatus* was able to complete development on the stems of *L*. *olgensis* and *P*. *jezoensis*. Egg-laying gallery length, adult female fecundity, larval gallery length, larval head capsule width, and adult brood weight were significantly higher on *L*. *olgensis* (**Figures 1A, B, D–F**), except that egg hatchability was significantly higher on *P*. *jezoensis* (**Figure 1C**). Although both bark beetles can develop on the stem of *P*. *jezoensis*, the adults of *I*. *typographus* had significantly higher fecundity and longer ovipositional gallery, and their larvae had significantly longer gallery length, wider head capsule, and higher adult brood weight than those of *I*. *subelongatus*, while the hatching rate of *I*. *subelongatus* was significantly higher than that of *I*. *typographus* (**Supplementary Table 1**).

### Lesion area and ergosterol amount in response to fungal inoculation

**Figure 2:**
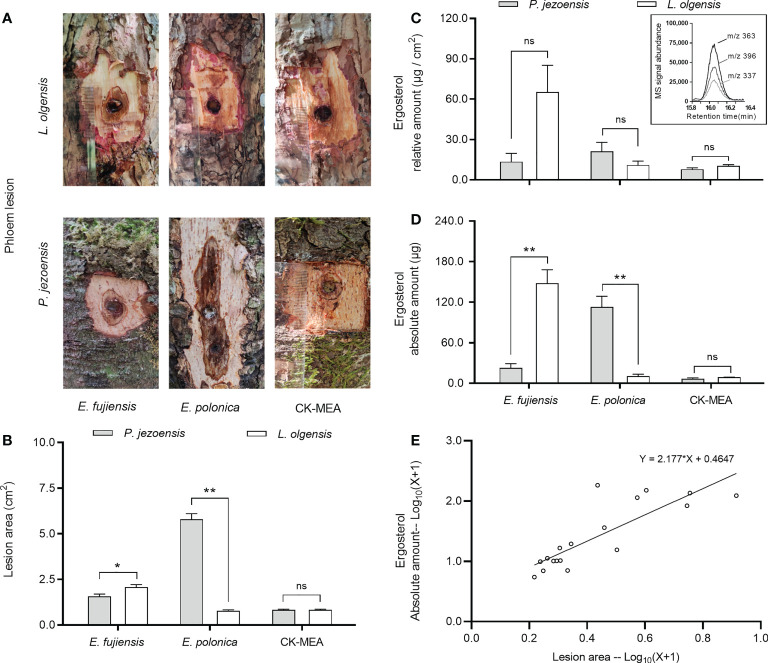
Mean ( ± SE) lesion areas and ergosterol on *P. jezoensis* and *L. olgensis* in response to culture inoculations. **(A)** Phloem lesion area of *E. fujiensis*, *E. polonica*, and MEA on *P. jezoensis* and *L. olgensis*, respectively; **(B)** lesion area, **(C)** relative amount of ergosterol and **(D)** absolute amount of *E. fujiensis*, *E. polonica*, and MEA on *P. jezoensis* and *L. olgensis*, respectively; **(E)** linear regression analysis between lesion area and absolute amount of ergosterol. Inset shows ion signal abundance of selective ions (m/z 337, 363, and 396) of ergosterol. Asterisks indicate significant differences (*p < 0.05, **p < 0.01, ns indicates no significant differences; T-test for independent samples, *α*=0.05; *n*=3 tree replicates per treatment).

*Endoconidiophora fujiensis* caused a significantly larger area of infection on the phloem of *L. olgensis* than on *P*. *jezoensis*, while the lesion area induced by *E. polonica* on *P. jezoensis* was significantly larger than on *L. olgensis* (**Figures 2A, B**). Regarding the relative amount of ergosterol produced, the two fungi did not show significant differences between two host trees, as did MEA culture inoculation (**Figure 2C**). For the absolute amount of ergosterol accumulated on the lesion, the significant differences between two host trees showed a general similarity with the above lesion areas (**Figure 2D**). We further analyzed the relationship between lesion area and the amount of accumulated ergosterol using linear regression and found that they were positively related (slope =2.177, *R^2^ =* 0.6925) (**Figure 2E**). Moreover, in *P*. *jezoensis, E*. *polonica* caused significantly larger lesion areas and more abundant absolute ergosterol amounts than *E*. *fujiensis*; however, in *L*. *olgensis*, *E*. *fujiensis* caused significantly larger lesion areas and more abundant ergosterol amounts (absolute and relative amount) than *E*. *polonica*; the relative amount of ergosterol produced by *E. polonica* was highest in *P. jezoensis*, but was not significantly different from treatment with *E. fujiensis* (**Supplementary Table 2**).

### Volatile monoterpenes in tree bark in response to fungal inoculation

**Figure 3:**
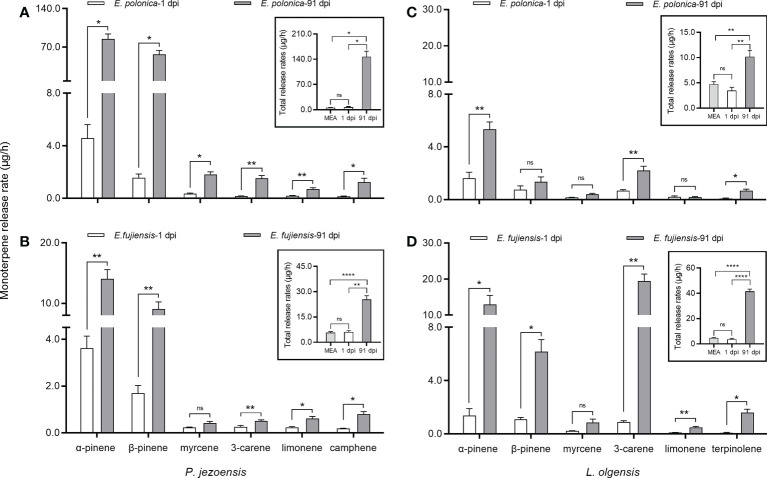
Mean ( ± SE) release rates (μg/h) of monoterpenes of *P. jezoensis* and *L. olgensis* in response to culture inoculations. **(A, C)** *P*. *jezoensis* and *L. olgensis* inoculated with *E. polonica* and MEA, respectively; **(B, D)** *P*. *jezoensis* and *L. olgensis* inoculated with *fujiensis* and MEA, respectively. Insets show total monoterpene release (μg/h) for each treatment. Asterisks indicate significant differences (*p < 0.05, **p < 0.01, ****p < 0.0001) and ns indicates no significant differences (T-test for independent samples, *α*=0.05; *n*=3 tree replicates per treatment).

The fungus is assumed to cause short-term induction of tree monoterpenes at 1 dpi, while long-term induction of tree monoterpenes is assumed to occur at 91 dpi. Regardless of 1 dpi or 91 dpi, the release rates of host monoterpenes induced by the MEA culture showed no differences from the healthy treatment on two host trees (**Supplementary Table 3**), so we summarize the data of each monoterpene induced by the MEA culture at 1 dpi and 91 dpi as a result. In the short term, no significant differences in the total amount of monoterpenes were observed between the amount induced by the fungal culture and the amount induced by the MEA culture (**Figure 3**, inset). However, in the long term, the fungal inoculations caused the release rate of total monoterpenes from *P*. *jezoensis* and *L*. *olgensis* to be significantly higher than from the MEA culture (**Figure 3**, inset).

Comparing the short- and long-term release of monoterpenes, we found that the release rate of total monoterpenes in *P*. *jezoensis* inoculated with *E*. *polonica* was 20-fold higher at 91 dpi than in *L*. *olgensis* inoculated with *E*. *polonica* (**Figures 3A, C**, inset); in contrast, the release rate of total monoterpenes in *L*. *olgensis* inoculated with *E*. *fujiensis* was 10-fold higher at 91 dpi than at 1 dpi, but it was 3-fold higher in *P*. *jezoensis* inoculated with *E*. *fujiensis* (**Figures 3B, D**, inset). The difference in the release rate of each volatile monoterpene between 1 dpi and 91 dpi also varied with each fungal treatment. In *P*. *jezoensis* inoculated with *E*. *polonica*, the release rate of each monoterpene was significantly higher at 91 dpi than at 1 dpi (**Figure 3A**). In *P*. *jezoensis* inoculated with *E*. *fujiensis*, only the release rate of myrcene showed no differences between 1 dpi and 91 dpi; the release rates of the other monoterpenes were significantly higher at 91 dpi (**Figure 3B**). In *E*. *polonica-*inoculated *L*. *olgensis*, the release rates of α-pinene, 3-carene, and terpinolene were significantly higher at 91 dpi, whereas the other monoterpenes showed no differences between 1 dpi and 91 dpi (**Figure 3C**). In *E*. *fujiensis-*inoculated *L*. *olgensis*, the release rates of myrcene at 91 dpi were not significantly different from those at 1 dpi, but the release rates of the other monoterpenes were all significantly higher at 91 dpi (**Figure 3D**).

Despite the different culture types, α-pinene had the highest release rate among the volatile monoterpenes in *P*. *jezoensis*, not only at 1 dpi but also at 91 dpi, followed by β-pinene (**Supplementary Table 3**). In *L*. *olgensis*, the release rate of α-pinene was highest in the fungal treatments at 1 dpi, while the release rate of 3-carene was highest in the control treatments; however, at 91 dpi, 3-carene had the highest release rate in the *E*. *fujiensis* treatment, while α-pinene still had the highest release rate in the *E*. *polonica* treatment (**Supplementary Table 3**).

### Phenolic compounds in tree bark in response to fungal inoculation

**Figure 4:**
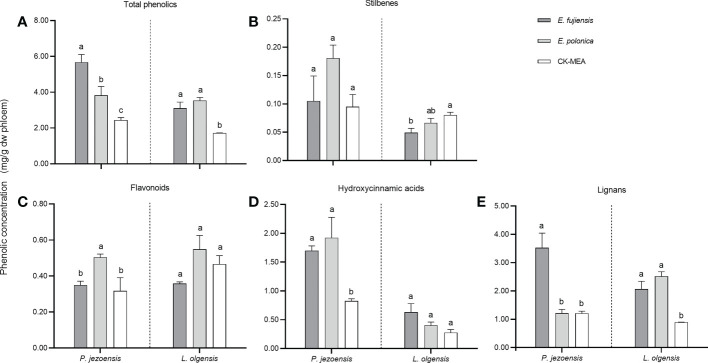
Mean (± SE) phenol concentrations (mg/g dw phloem) in the phloem of *P. jezoensis* and *L. olgensis* in response to culture inoculations. **(A)** Total phenols; **(B–E)** phenolic class stilbenes, flavonoids, hydroxycinnamic acids, and lignans, respectively. Different letters in each graph indicate significant differences (Turkey HSD test, a=0.05; n=3 tree replicates per treatment).

A total of ten phenolic compounds belonging to four classes (stilbenes, hydroxycinnamic acids, flavonoids and lignans) were detected in both healthy and culture-inoculated phloem of *P. jezoensis* and *L. olgensis* (**Supplementary Table 4**). In general, total concentrations of phenols induced by the fungi were significantly higher than those induced by the MEA culture; at the same time, *E. fujiensis* induced significantly higher phenols than *E. polonica* in *P. jezoensis*, whereas no significant differences were detected between the two fungi in *L. olgensis* (**Figure 4A**); total concentrations of phenols induced by the culture inoculations were significantly higher than those induced by the healthy treatments in each tree (**Supplementary Table 4**).

To compare the differences in phenols induced by two fungi, we use MEA culture inoculations as control. As for the class of stilbenes, the induced concentration for *P. jezoensis* did not differ among the three treatments, whereas for *L.olgensis* it was highest in the MEA culture and significantly higher than the *E. fujiensis* treatment, while it was not significantly different from the *E. polonica* treatment (**Figure 4B).** For the class of flavonoids, *E. polonica* induced the highest level on *P. jezoensis*, followed by the treatments with *E. fujiensis* and MEA, both of which were significantly lower than *E. polonica* and did not differ from each other; no significant differences in concentration were observed among the three treatments for *L. olgensis* (**Figure 4C**). For the class of hydroxycinnamic acids, in *P. jezoensis*, the concentrations induced by two fungi were both significantly higher than those of MEA controls, at the same time, no significant differences were found between two fungal treatments; in *L. olgensis*, the concentration induced by *E. fujiensis* was the highest, but was not significantly different from the concentration induced by *E. polonica* and MEA treatments (**Figure 4D**). For the class of lignans, the concentration induced by *E. fujiensis* was significantly higher than that of *E. polonica* and MEA for *P. jezoensis*, with no significant differences detected between the latter two treatments; for *L. olgensis*, the concentrations induced by the two fungal treatments were not different from each other, but both were significantly higher than those of the MEA culture (**Figure 4E**).

### Lignin monomers of G6 and G4 in response to fungal inoculation

**Figure 5:**
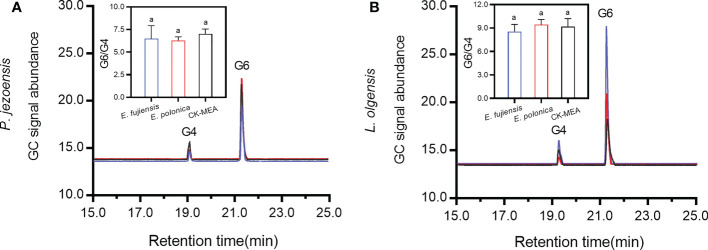
Chromatograms of 3,4-dimethoxybenzldehyde (G4) and 3,4-dimethoxybenzoic acid (G6) in *P. jezoensis* **(A)** and *L. olgensis* **(B)** in response to culture inoculations. Inset plots show the mean (± SE) ratio of peak area of G6 to G4. Different letters in each inset plot indicate significant differences (Turkey HSD test, *α*=0.05; *n*=3 tree replicates per treatment).

The sapwood samples of the culture-inoculated lesions of *P. jezoensis* and *L. olgensis* (guaiacyl lignin) were analyzed by TMAH thermochemolysis/GC (**Figure 5**). For *P. jezoensis*, no significant differences were found in the ratio of G6 to G4 between *E. fujiensis*-inoculated, *E. polonica*-inoculated, and CK-MEA-inoculated sapwood lesions (**Figure 5A**). For *L. olgensis*, there were also no significant differences in the ratio of G6 to G4 among the three treatments (**Figure 5B**). The mean ratio of G6 to G4 ranged from 8.55 to 9.42 for *L. olgensis*, which was higher than for *P. jezoensis* (ranged from 6.29 to 7.02) (**Figure 5**).

### Correlation of biochemical responses of trees and fungi at 91 dpi

**Figure 6:**
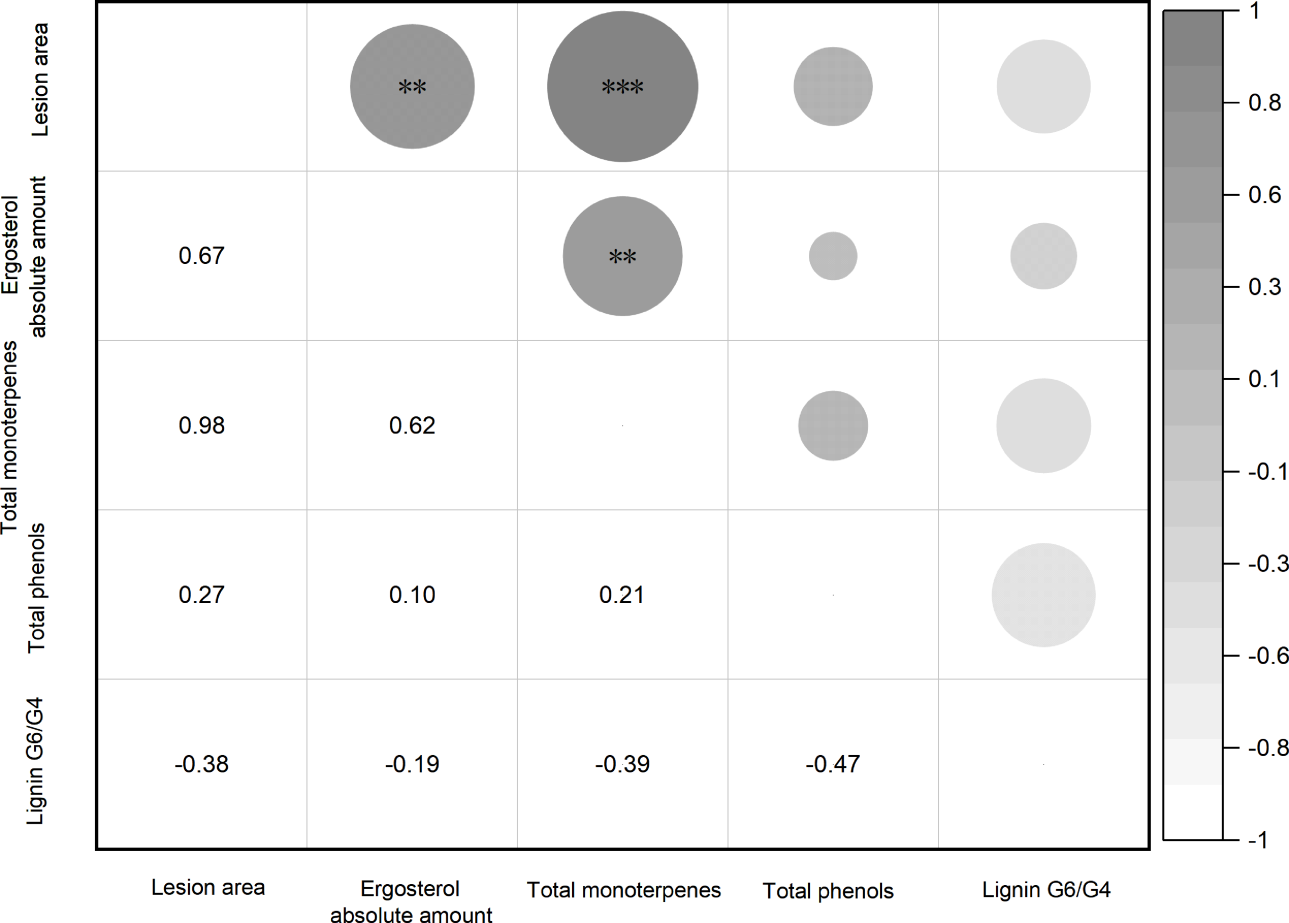
Correlation analysis for lesion area, ergosterol amount, total monoterpene, total phenols, and ratio of lignin G6 to G4 at 91 dpi. The number in the lower triangle represents the Pearson *r* value and the significant marker in the upper triangle represents the *P* range (**p< 0.01, ***p < 0.001; Pearson correlation, *α*=0.05; *n*=18 replicates per treatment).

To analyze the correlations between fungal infection and tree biochemical responses, the correlation matrix for lesion area and ergosterol amount, total monoterpene release, total phenols and lignin G6/G4 at 91 dpi was performed with the experimental data of all culture inoculations. As shown in **Figure 6**, total monoterpene release correlated strongly with lesion area (Pearson *r* =0.98, *P* value < 0.0001) and ergosterol amount (Pearson *r* =0.62, *P* value =0.0061); total phenols did not correlate with lesion area (Pearson *r* =0.27, *P* value =0.2800) and ergosterol amount (Pearson *r* =0.10, *P* value =0.6927); lignin G6/G4 also did not correlate with lesion area (Pearson *r* =-0.38, *P* value =0.1188) and ergosterol amount (Pearson *r* = -0.19, *P* value =0.4435).

## Discussion

### Two bark beetles performance on host trees

Although two bark beetles are capable of colonizing and developing well on the stems of *P. jezoensis*, *I. typographus* showed significantly better suitability than *I. subelongatus* in terms of developmental indices such as fecundity of adult females, length of larval galleries, weight of adult brood, etc. However, on the trunks of *L. olgensis*, *I. subelongatus* successfully completed reproduction and brood development, while some adults of *I. typographus* excavated only a short tunnel in the phloem, some gathered in an invasion hole, and all did not survive. The above biological developmental indices suggest that *I. subelongatus* can use host trees of *I. typographus*, but not vice versa, which is consistent with the observations in the field (Xiao, 1992). The differences in suitability of the two bark beetles on their respective host trees is probably related to the available food in the phloem and the degree of stiffness of the outer bark of the two host trees (Wainhouse et al., 1990). While our study helps evaluate the relative differences in reproductive and developmental potential of two bark beetles on two host trees, it remains unclear how induced phytochemical defense affects the fitness of two bark beetles in host trees, because studies on living trees in this system are not possible (Cale et al., 2015).

### Two associated fungal infections on host trees

Using the phloem of the host tree as “culture medium”, the lesion area was examined for its suitability of fungal growth. *E. polonica*, which is associated with *I. typographus*, cannot grow on the phloem of *L. olgensis*, but grows well on *P. jezoensis.* However, *E. fujiensis* associated with *I. subelongatus* can grow on both *L. olgensis* and *P. jezoensis* and grows much better on the former. This infection of two fungi on two host trees is consistent with previous findings that *E. fujiensis* and *E. polonica* (formerly *C. fujiensis* and *C. polonica*) can cause significantly larger sapwood lesions only on *Larix kaempferi* and *Picea abies*, respectively (Harrington et al., 2002). Comparing the growth characteristics of two fungi and the developmental characteristics of two bark beetles on two tree species, we find that *E. polonica* and *I. typographus* respond convergently to *P. jezoensis*, and *E. fujiensis* and *I. subelongatus* respond convergently to *L. olgensis*.

Usually, lesion length is used as an indicator of virulence, and longer lesions are thought to be caused by more pathogenic partners (Matsuya et al., 2003; Rice et al., 2007; Fang et al., 2020a). In our study, lesion length is replaced by lesion area, so a larger lesion area indicates higher virulence. According to this line, *E. fujiensis* was more pathogenic to *L. olgensis*, while *E. polonica* was more pathogenic to *P. jezoensis*. A more virulent fungal partner of a bark beetle would be more beneficial because it likely contributes to tree mortality (Solheim and Safranyik, 1997; Viiri and Lieutier, 2004). The difference in virulence of *E. fujiensis* and *E. polonica* for two tree species strongly suggests that they are beneficial to their respective beetle vectors, indirectly confirming that the specific associations occur in the “*E. fujiensis-I. subelongatus*” and “*E. polonica-I. typopraphus*” complexes. These differences are also consistent with previous findings that *E. polonica* and *E. fujiensis* differ primarily in their physiological specialization on the hosts of their bark beetle vectors (Harrington et al., 2002). Coincidentally, *E. fujiensis* and *E. polonica* have been shown to be the most pathogenic fungi associated with two bark beetles (Solheim, 1986; Solheim, 1992a; Solheim, 1992b; Yamaoka et al., 2009), and both are capable of killing host trees at high inoculation concentrations (Yamaoka et al., 1998; Yamaoka et al., 2000). The damage caused by forest insect symbioses is often unexpected and unpredictable, making management of these associations very difficult (Ploetz et al., 2013; Wingfield et al., 2016).

### The response of associated fungi to host trees

#### Associated fungi produce a lot of ergosterol on the host trees

Ergosterol is not produced by plants or insects but is an important component of fungal cell membranes for maintaining integrity and fluidity (Guevara-Rozo et al., 2020). Fungal species capable of producing large amounts of ergosterol could provide abundant food for symbiotic beetle larvae and further promote beetle larval development on host trees. For example, ergosterol provided by associated fungi (e.g., *Ophiostoma montium*) is likely the primary sterol source for larvae of *Dendroctonus ponderosae* Hopkins (Six and Paine, 1998; Guevara-Rozo et al., 2020). Ergosterol content is commonly used as a proxy for determining fungal biomass in a substrate (Porep et al., 2014) or as an indicator of fungal growth rate (Bentz and Six, 2006; Guevara-Rozo et al., 2020). In this study, the relative amount of ergosterol produced by *E. fujiensis* was not significantly different on two host plants, suggesting that the infection rate of *E. fujiensis* is similar on two host trees; *E. polonica* also showed a similar pattern to *E. fujiensis* on two host trees. Interestingly, the ergosterol content produced by *E. polonica* was the highest in *P. jezoensis*, with no significant differences from the *E. fujiensis* treatments, while the ergosterol content produced by *E. fujiensis* was the highest in *L. olgensis*, significantly higher than in the *E. polonica* treatments. On the one hand, this strongly suggests that the host substrate play an important role in the spread of the fungus. Previous reports have indicated that ergosterol content in the fungal membrane may allow plants to identify a fungus as a potential pathogenic organism (Cardoza et al., 2015; Malmierca et al., 2015). On the other hand, this suggests that the infectivity of *E. fujiensis* is similar to that of *E. polonica* on *P. jezoensis*. As in previous studies where low ergosterol levels were found in uninfected phloem samples and MEA-inoculated trees (Guevara-Rozo et al., 2020), low ergosterol levels were also found in CK-MEA inoculated *L. olgensis* and *P. jezoensis*, suggesting the presence of fungal endophytes (Ganley et al., 2004; Giordano et al., 2009). Coincidentally, three plant sterols, campestetol, β-sitosterol, and sitostenone, were found in the phloem of two host trees and identified with standard chemicals; however, the amount of ergosterol is relatively low compared to the three host sterols (**Supplementary Figure 1**).

#### Associated fungi cause the release of large amounts of monoterpenes in the host tree

Fungal activity in the phloem is critical because the induced defense mechanisms are initiated and formed in the phloem of the tree (Six and Wingfield, 2011). There is ample evidence that monoterpenes are characterized by fungicidal activities in tree defense (Cale et al., 2017; Fang et al., 2020a). In this study, there were significantly higher release of total monoterpenes at 91 dpi compared to 1 dpi in two fungal treatments, indicating the potential risk of an arms race between fungi and tree hosts (Franceschi et al., 2005). Interestingly, the release rate of total monoterpenes from *P*. *jezoensis* inoculated with *E*. *polonica* and *L*. *olgensis* inoculated with *E*. *fujiensis* increased sharply at 91 dpi compared to 1 dpi, whereas there was little increase in *P*. *jezoensis* inoculated with *E*. *fujiensis* and *L*. *olgensis* inoculated with *E*. *polonica* at 91 dpi compared to 1 dpi. These results support the idea that more pathogenic fungi can induce a stronger defense response in host trees (Solheim, 1992b; Krokene and Solheim, 1996). The pathogenic fungi stimulate defenses in the phloem of the tree, ultimately depleting them and making it easier for bark beetle vectors to invade the trees (Six and Wingfield, 2011). However, the two fungi did not induce monoterpene defense of trees in the short term compared with two control treatments, contradicting the report of Fang et al. (2020a).

In the study, we identified the major components of monoterpenes on two host trees based on the release rate greater than 0.05 μg/h and the 100% detected rate of monoterpenes in each treatment. Different cultural inoculation treatments result in different levels of monoterpene release in two host trees. In *P. jezoensis*, the release rate of α-pinene was always highest, whether inoculated or not, followed by β-pinene, which is consistent with previous observations in spruce phloem (Zhao et al., 2011; Netherer et al., 2021). Moreover, the release rates of these two monoterpenes in *P. jezoensis* increased more when treated with *E. polonica* at 91 dpi compared to 1 dpi than when treated with *E. fujiensis*. However, in *L. olgensis*, α-pinene was not always highest in all treatments, and sometimes replaced by β-pinene and 3-carene; furthermore, the release rates of α-pinene and β-pinene increased more when treated with *E. fujiensis* at 91 dpi compared with 1 dpi than when treated with *E. polonica*. We can see the specific and differential increases in the release rate of certain monoterpenes induced by two fungi on two host trees at 91 dpi compared to 1 dpi.

α-Pinene exhibits growth retarding and inhibitory effects on *E. polonica* and *E. fujiensis in vitro* (unpublished data), exerts a significant behavioral inhibitory effect on mass attacks of *I. subelongatus* (Fang et al., 2020a), but shows a significant behavioral synergistic effect on the pheromone source of *I. typographus* (Fang et al., 2020b). Importantly, *S*-(-)-α-pinene can be directly converted to 4*S*-*cis*-(-)-verbenol, which is one of the two major components of the aggregation pheromone of *I. typographus* (Schlyter et al., 1987; Schlyter et al., 1992). It can be concluded that the release of α-pinene during treatment with *E. polonica* helps *I. typographus* better attack *P. jezoensis*, while inhibiting the invasion of *I. subelongatus* and the mycelial growth of *E. fujiensis*. 3-Carene suppresses the growth of *E. fujiensis* but shows a strong synergistic behavioral effect on the catches of *I. subelongtus* with pheromone sources (Fang et al., 2020a). However, the increase in the release rate of 3-carene at 91 dpi compared to 1 dpi was less than that of α-pinene in *L. olgensis* inoculated with *E. polonica*, suggesting that the attractive effect of 3-carene on *I. subelongtus* is suppressed by α-pinene, making it unlikely that *E. polonica* assists *I. subelongtus* in attacking *L. olgensis*. Erbilgin (2018) concluded that phytochemicals serve as mediators for the expansion of the host range of *Dendroctonus ponderosae*. In general, these two pathogenic fungi affected some phytochemicals of host trees, mediated the arms race between beetles and hosts, and thus influenced the expansion of the host range of the two bark beetles.

The change in the rate of release of each monoterpene between long-term and short-term infestations of the same tree was strongly dependent on the fungal species. For example, in *L. olgensis*, the release rate of limonene was significantly increased after 91 dpi compared to 1 dpi when treated with *E. fujiensis*, whereas it did not change significantly between 91 dpi and 1 dpi when treated with *E. polonica*. This phenomenon was also observed in *Larix principis-rupprechtii* inoculated with two strains of *E. fujiensis* (Fang et al., 2020a). A possible explanation for this would be the specificity of the fungus, since the inoculated hosts are the same species with relatively similar physiological conditions and habitats. It should be noted that infestation of trees with pathogenic fungi would result in each monoterpene component acting synergistically and making up most of the tree’s defense system, so we need to pay more attention to the overall dynamics of monoterpenes before and after fungal inoculation. In addition to monoterpenes, other phytochemicals such as diterpenes in the phloem are toxic to many fungal partners of bark beetles (Erbilgin et al., 2016; Cale et al., 2019), so further studies should be conducted to comprehensively analyze defense responses against pathogenic fungi.

#### Associated fungi induce an increase in phenols on the lesion of host trees

In two host trees, both of two fungi induced significantly higher concentrations of total phenols within the lesion than the MEA culture, suggesting that phenols induce resistance to pathogenic fungi (Brignolas et al., 1995; Evensen et al., 2000). However, this result is inconsistent with the finding that the total amount of phenols in the *Grosmannia clavigera* infected lesion was lower than in the jack pine wound control (Klutsch et al., 2017). Erbilgin et al. (2016) found that fungal infection decreased phenol production in lodgepole pine, whereas phenols in jack pine did not change in response to fungal infection. It is possible that differences between tree species and other factors influence phenol accumulation on the lesion, so further studies are needed to clarify the role of phenols against fungi. *E. fujiensis* induced more phenolics produced on *P. jezoensis* than *E. polonica*; however, on *L. olgensis*, the total amount of phenols induced by *E. polonica* was higher than that induced by *E. fujiensis*, indicating the opposite effects of host tree defenses against species-specific fungi. Terpenes and phenols are considered to be C-based defense chemicals (Keeling and Bohlmann, 2006), and therefore compete for carbon reserves in the lesion during the synthesis phase. Monoterpenes and phenols are biosynthesized by different pathways. Monoterpenes are preferentially produced in plants *via* the 2*C*-methyl-D-erythritol-4-phosphate pathway (Bohlmann et al., 1998), whereas phenols are biosynthesized *via* the phenylpropanoid pathway (Boudet et al., 1995). Differences in the distribution of carbohydrates between these two classes may influence their production (Klutsch et al., 2017). Erbilgin et al. (2016) found that increases in monoterpenes and decreases in phenols in the lesion tissue are important determinants of host suitability for tree physiology and thus contribute to invasion success of forest insects. The situation described above proves that *P. jezoensis* infected with *E. polonica* and *L. olgensis* infected with *E. fujiensis* were more suitable for invasion by *I. typographus* and *I. subelongatus*, respectively. A comparison of the correlation between fungal infection and host defense responses revealed a strong correlation for total monoterpenes but not for total phenols. This is consistent with the reports of Erbilgin et al. (2016). Despite some correlation between the production of terpenes and phenols in response to fungal infection, phenols did not clearly determine the host suitability for fungi.

A total of four classes of phenols were detected in two host trees, whether or not a cultured fungus was inoculated, with phenolic components similar to those of lodgepole pine and whitebark pine (Raffa et al., 2017 **)** and more diverse but less abundant than monoterpenes in two host trees. Most phenols can be converted to other compounds by fungi (Hammerbacher et al., 2013), so their concentrations varied with the duration of fungal infection. Among the phenol classes, stilbenes typically have potent antifungal activity (Hammerbacher et al., 2013) and showed a marked increase in locally induced tissues in two pines (Raffa et al., 2017). In our study, stilbenes induced on *P. jezoensis* did not differ from fungi and MEA control, which is a deviation from the above reports. Moreover, flavonoids and hydroxycinnamic acids induced on *L. olgensis* followed the same general pattern among the three treatments. Previous studies have reported that the flavonoids catechin and the hydroxycinnamic acids taxifolin have inhibitory effects on bark beetle associates (Hammerbacher et al., 2019). Because we did not take multiple samples during the course of the experiment, the dynamic change in phenols was not recorded. We do not know how their concentration changes during fungal infection, but it is certain that some components of the phenol class are important for host resistance to fungi and also serve as antifeedant semiochemicals for bark beetles (Faccoli and Schlyter, 2007). With the exception of *L. olgensis* stilbene, two fungi caused a greater or lesser increase in concentrations of other phenols in hosts and showed a similar pattern to total phenols in response to fungal infection.

#### Associated pathogenic fungi cannot cause side-chain oxidation of host tree lignin structure

Two *Ips* bark beetles can excavate the nuptial chambers or tunnel galleries deep into the sapwood, indicating their ability to destroy lignin. As the insects feed on the relatively stiff structures of the plant, wear of the upper jaw increases more dramatically (Raupp, 1985). Basidiomycetes are considered the only fungi associated with bark beetles that can degrade lignin (Six and Klepzig, 2021), making this the first study to confirm the ability of pathogenic fungi to degrade lignin using TMAH thermochemolysis. In this study, *E. fujiensis* and *E. polonica* do not significantly promote side-chain oxidation of lignin degradation despite tree species, which is consistent with the fact that ascomycete fungi cannot degrade lignin (Six, 2020). Compound G4 is thought to result from TMAH thermochemolysis of those β-O-4 bonds that contain adjacent hydroxyl groups on the propyl side chain (Filley et al., 2000), and it should be the predominant product in undegraded wood (Geib et al., 2008). The average ratio of G6 to G4 was above 6.0 at CK-MEA, the mechanical damage control, suggesting that G4 was the less predominant product in undecomposed wood. A plausible explanation could be that the thermochemolysis temperature of the process (240°C in the study) was lower than in other studies (250°C), resulting in a relatively lower yield of G4.

It has been suggested that the lack of consistency of association between a bark beetle and a single fungus, which has high efficacy in stimulating defenses, may be offset by the fungal complexes that a bark beetle often carries (Six and Wingfield, 2011), since most bark beetles are associated with a complex of fungal species (Kirisits, 2004). While *E. polonica* and *E. fujiensis* are the highly pathogenic fungi associated with *I. typographus* and *I. subelongatus*, respectively, the consistent association of the “*E. polonica*-*I. typographus”* and “*E. fujiensis*-*I. subelongatus”* complexes appears to be lacking due to the highly random occurrence of the two fungi (Persson et al., 2009; Yamaoka et al., 2009). However, our study provides direct (ergosterol) and indirect (release of monoterpenes) evidence that the “benefits” of the two fungi are specific and different for their respective beetle vectors. Furthermore, both ergosterol levels and total monoterpene release correlated with lesion area, indicating the important role of fungal pathogenicity in supporting beetle vectors.

## Conclusion

In summary, our study provides the first information on the consistent suitability of bark beetles and associated pathogenic fungi on host plants based on biochemical experiments. The “*I. typographus*-*E. polonica”* complex showed good suitability on *P. jezoensi* but cannot colonize on *L. olgensis*; the “*I. subelongatus-E. fujiensis”* complex performed well on *L. olgensis* and also showed some suitability on *P. jezoensis*. The “*I. subelongatus-E. fujiensis”* complex also showed borderline host suitability and requires attention to expand the host range in the future. All aggressive bark beetles are associated with relatively pathogenic fungi, and these fungi tend to be relatively vector-specific (Franceschi et al., 2005). Our study also confirmed species-specific association relationships in the “*I. typographus*-*E. polonica”* complex and the “*I. subelongatus-E. fujiensis”* complex. An interesting correlation between monoterpene release and fungal infection was found in host phloem responses to fungal species. The virulence of the fungi probably itself contributes to more severe infestation of the tree by providing more food for the vector beetle and placing greater demands on the defense of the host tree. We also demonstrated that monoterpenes are more important than phenols for host tree defenses against two pathogenic fungi and are an important indicator of host suitability for pathogenic fungal infection.

## Data availability statement

The original contributions presented in the study are included in the article/**Supplementary Material**. Further inquiries can be directed to the corresponding author.

## Author contributions

XK and XS conceptualized and designed the research. XS, JF, and HD performed the experiments. XS analyzed the data and wrote the manuscript. XS, JF, SZ, FL, ZZ and XK participated in the discussion for experimental details. XK directed the project and revised the manuscript. All authors contributed to the article and approved the submitted version.

## Funding

This work was funded by the Fundamental Research Funds of the Chinese Academy of Forestry (project no. CAFYBB2017ZB002), and partial financial support was received from the Demonstration and Extension of Biocontrol Technology for Bark Beetles in Natural Spruce Forest in Qinghai (project no. Qing2021-TG06).

## Acknowledgments

We thank Zheng Wang (Chinese Academy of Forestry, Beijing) for providing experimental fungal species, Xueqiang Wang (Wangqing Forestry Bureau, Jilin Province) and Desheng Zhong (Mengjiagang Forestry Farm, Heilongjiang Province) for bark beetle collection and fieldwork support.

## Conflict of interest

The authors declare that the research was conducted in the absence of any commercial or financial relationships that could be construed as a potential conflict of interest.

## Publisher’s note

All claims expressed in this article are solely those of the authors and do not necessarily represent those of their affiliated organizations, or those of the publisher, the editors and the reviewers. Any product that may be evaluated in this article, or claim that may be made by its manufacturer, is not guaranteed or endorsed by the publisher.

## Supplementary material

Supplementary Figure 1 The amounts of three plant sterols detected in the phloem of two host trees.
