# Supplementary figures and images for: Performance of two Ips bark beetles and their associated pathogenic fungi on hosts reflects a species-specific association in the beetle-fungus complex
Source: Front Plant Sci. 2022 Nov 22;13:1029526. doi: 10.3389/fpls.2022.1029526 (PMC9722963; doi:10.3389/fpls.2022.1029526)

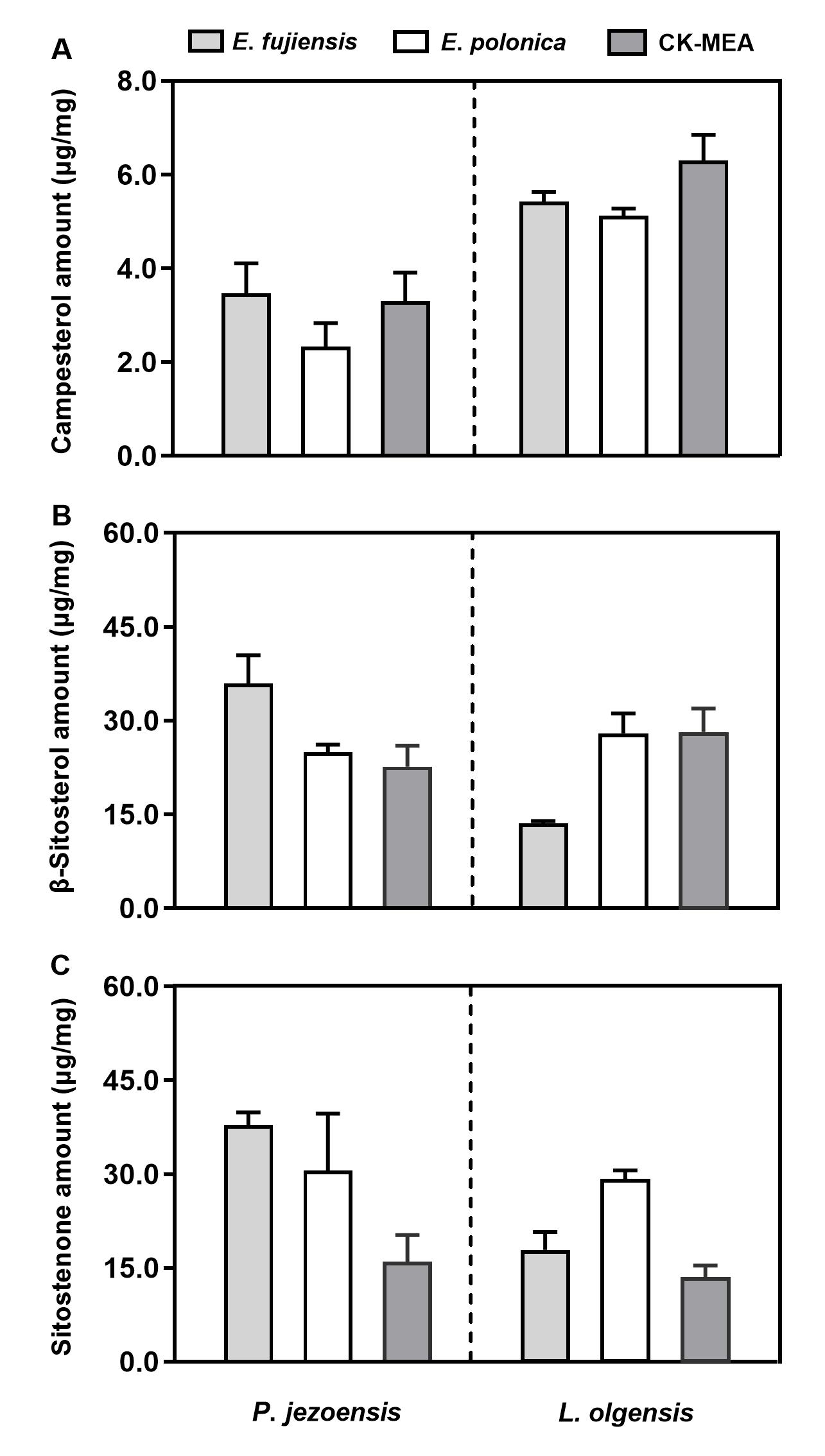

Supplement: Supplementary Figure 1 — The amounts of three plant sterols detected in the phloem of two host trees. [file Image_1.tif]
